# Beyond Biocompatibility: Immune Dysregulation, Oxidative Stress, and Tissue Intolerance Associated with Ti-6Al-4V Dental Implants—A Critical Review and Perspective

**DOI:** 10.3390/antiox15030365

**Published:** 2026-03-13

**Authors:** Żaneta Anna Mierzejewska, Łukasz Woźniak, Jérôme R. Lechien, Jan Borys, Kamila Łukaszuk, Bożena Antonowicz

**Affiliations:** 1 Institute of Biomedical Engineering, Faculty of Mechanical Department, Bialystok University of Technology, Wiejska 45C, 15-351 Bialystok, Poland; 2 Department of Dental Surgery, Medical University of Bialystok, M. Sklodowskiej-Curie 24A, 15-276 Bialystok, Poland; bozena.antonowicz@umb.edu.pl; 3 Department of Surgery, UMONS Research Institute for Health Sciences and Technology, University of Mons, Place du Parc 20, 7000 Mons, Belgium; jerome.lechien@umons.ac.be; 4 Department of Otolaryngology-Head & Neck Surgery, Foch Hospital, Paris Saclay University, 921500 Suresnes, France; 5 Department of Maxillofacial and Plastic Surgery, Medical University of Bialystok, M. Sklodowskiej-Curie 24A, 15-276 Bialystok, Poland; jan.borys@umb.edu.pl (J.B.); kamila.lukaszuk@umb.edu.pl (K.Ł.)

**Keywords:** dental implants, titanium alloys, Ti-6Al-4V, oxidative stress, immune response, peri-implantitis, metal intolerance, inflammation, biocompatibility

## Abstract

Titanium and its alloys are widely used in dental implantology due to their favorable mechanical properties and well-documented long-term clinical performance. Among them, Ti-6Al-4V is particularly common in load-bearing applications. Nevertheless, a growing body of experimental and clinical evidence suggests that Ti-6Al-4V implants cannot be regarded as biologically inert in all patients. Adverse tissue responses, such as impaired healing, chronic peri-implant inflammation, and unexplained implant failure, have been reported even in the absence of classical risk factors, including infection, mechanical overload, or confirmed metal allergy. This critical review challenges the prevailing assumption that these complications are driven primarily by mechanical or immunoallergic mechanisms. Instead, oxidative stress is proposed as a central and unifying factor underlying adverse tissue reactions to Ti-6Al-4V dental implants. Corrosion, tribocorrosion, and mechanical wear lead to the release of titanium-, aluminum-, and vanadium-containing particles and ions, which promote excessive generation of reactive oxygen species at the implant–tissue interface. The resulting redox imbalance disrupts bone remodeling, impairs osteogenic differentiation, and maintains a pro-inflammatory microenvironment. Importantly, pathology arises not merely from increased reactive oxygen species production, but from the failure of local antioxidant defense systems to counteract this burden. Insufficient enzymatic and transcriptional antioxidant responses result in persistent redox imbalance, sustained innate immune activation, and progressive tissue intolerance. Oxidative stress is therefore conceptualized not as a secondary byproduct of inflammation, but as a primary driver of immune dysregulation through chronic macrophage activation and inflammasome signaling. This redox-driven feedback loop amplifies tissue damage and compromises long-term osseointegration independently of classical adaptive immune sensitization. Recognizing oxidative stress as a key determinant of implant–tissue interactions offers a more coherent framework for understanding implant-related complications and underscores the need for redox-aware biomaterial strategies and individualized patient risk assessment.

## 1. Introduction

Titanium-based dental implants are widely used in contemporary implant dentistry and are generally associated with high long-term survival rates. Their clinical success has traditionally been attributed to favorable mechanical properties, corrosion resistance, and the ability to achieve direct bone–implant contact through osseointegration. As a result, titanium has long been regarded as a paradigmatic example of a biocompatible and biologically inert biomaterial, a concept that continues to shape both clinical decision-making and biomaterials research [1,2,3]. However, the assumption that biocompatibility implies biological neutrality has increasingly been challenged. A growing body of experimental and clinical observations indicates that titanium implants, particularly those manufactured from the Ti-6Al-4V alloy, can actively interact with the host biological environment [4]. Clinical complications such as impaired soft tissue healing, persistent peri-implant inflammation, and progressive bone loss—hallmarks of peri-implantitis, a biofilm-associated pathological condition characterized by inflammation of peri-implant soft tissues accompanied by progressive loss of supporting bone beyond initial physiological remodeling—and unexplained early or late implant failure have been repeatedly reported in a subset of patients, even in the absence of overt infection, mechanical overload, or poor oral hygiene [5,6,7]. These findings suggest that factors beyond classical surgical or microbiological paradigms may contribute to adverse implant outcomes.

One emerging and underappreciated factor in this context is the disturbance of redox homeostasis at the implant–tissue interface. Titanium implants are not static entities; mechanical wear leads to the release of metal particles and ions during clinical service [8]. These degradation products, including titanium as well as alloying elements such as aluminum and vanadium in Ti-6Al-4V, possess the capacity to promote reactive oxygen species (ROS) generation in surrounding tissues [9,10]. Importantly, oxidative stress is not merely a byproduct of inflammation but a potent biological regulator that influences wound healing, bone remodeling, and immune cell behaviour [11]. Oxidative stress in peri-implant tissues therefore reflects not only excessive ROS generation, but also insufficient antioxidant buffering capacity. When antioxidant defense systems fail to adapt to continuous material-derived redox challenges, redox imbalance becomes sustained and biologically pathogenic rather than transient and adaptive.

Physiological levels of ROS play an essential role in normal tissue repair and osseointegration. However, excessive or sustained ROS production disrupts osteogenic differentiation, enhances osteoclast activity, and degrades extracellular matrix components, thereby shifting the balance from regeneration toward chronic tissue damage. Moreover, oxidative stress directly modulates immune responses at the implant site [12]. Macrophages, which are key orchestrators of implant integration, are highly sensitive to redox cues. A persistent oxidative environment favors pro-inflammatory macrophage activation, inflammasome signaling, and cytokine release, establishing a self-perpetuating cycle of inflammation and tissue degradation [13].

In this redox-centered framework, immune dysregulation should be viewed largely as a downstream consequence of oxidative imbalance rather than as an independent primary driver. Accordingly, in this review the term implant intolerance is used to describe a redox-driven failure of tissue adaptation to implant-derived material stressors, characterized by sustained oxidative imbalance, innate immune activation, and impaired regenerative responses, occurring independently of classical adaptive immune sensitization or confirmed metal allergy. This perspective helps to explain why many adverse reactions to titanium implants cannot be adequately accounted for by classical delayed-type hypersensitivity mechanisms. These mechanisms may explain implant failure in clinically well-maintained sites. Although such reactions are often labeled as “titanium allergy”, true antigen-specific adaptive immune responses to titanium appear to be rare and inconsistently supported by diagnostic testing. Framing implant-related complications as allergic phenomena therefore risks oversimplifying the underlying biology and obscuring more relevant pathogenic pathways.

**Figure 1:**
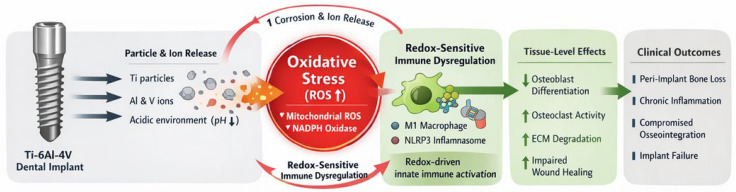
Oxidative stress–centered mechanisms underlying adverse tissue responses to Ti-6Al-4V dental implants. Corrosion, tribocorrosion, and mechanical wear of Ti-6Al-4V implants lead to the release of titanium-, aluminum-, and vanadium-containing particles and ions into peri-implant tissues. These degradation products promote excessive reactive oxygen species (ROS) generation at the implant–tissue interface, resulting in redox imbalance. Oxidative stress acts as a primary driver of immune dysregulation by sustaining pro-inflammatory macrophage activation and inflammasome signaling. Redox-sensitive immune responses impair bone remodeling, disrupt extracellular matrix homeostasis, and compromise soft tissue healing. Importantly, oxidative stress further accelerates material degradation, establishing a self-amplifying pathological feedback loop that ultimately destabilizes osseointegration and contributes to implant failure. Created in BioRender. Mierzejewska, A. (12 February 2026) https://BioRender.com/n92mqm4.

The aim of this critical review is to re-evaluate adverse tissue responses to Ti-6Al-4V dental implants through the lens of redox biology. We propose that oxidative stress represents a central and unifying mechanism that links material degradation, immune dysregulation, and impaired tissue integration (Figure 1). By synthesizing current experimental and clinical evidence, this article seeks to challenge the paradigm of universal titanium biocompatibility and to highlight the importance of redox-aware approaches in biomaterial selection, surface engineering, and patient-specific risk assessment.

This critical review was based on a structured literature search conducted in PubMed, Web of Science, and Scopus databases up to January 2026. Search terms included combinations of “titanium dental implants,” “Ti-6Al-4V,” “oxidative stress,” “reactive oxygen species,” “peri-implantitis,” “immune response,” “macrophage polarization,” and “corrosion products.” Experimental, clinical, and translational studies addressing redox mechanisms, material degradation, and immune modulation were included. Reviews, in vitro studies, animal models, and human investigations were considered, while purely mechanical or prosthetic studies without biological relevance were excluded. No formal systematic review protocol was applied, as the aim was to provide a critical mechanistic synthesis rather than quantitative meta-analysis.

## 2. Ti-6Al-4V Dental Implants as Sources of Oxidative Stress in Peri-Implant Tissues

### 2.1. Clinical Manifestations of Implant-Associated Tissue Reactions

Although titanium-based dental implants demonstrate high overall success rates, a subset of patients develops adverse tissue reactions that cannot be satisfactorily explained by mechanical overload, surgical trauma, or inadequate plaque control [14]. Clinically, these manifestations include delayed or impaired soft tissue healing, persistent peri-implant mucositis, progressive marginal bone loss, and early or late implant failure. In some cases, local symptoms are accompanied by extraoral or systemic complaints, such as nonspecific inflammatory symptoms or cutaneous reactions, following implant placement [14,15,16,17]. Importantly, such complications are frequently observed in patients without classical risk factors for peri-implant disease. Unlike periodontitis, these reactions may occur under low plaque scores. This discrepancy suggests that implant-related failures cannot always be reduced to technical or microbiological causes alone [18,19]. Instead, increasing attention has been directed toward biological processes occurring at the implant–tissue interface, particularly those capable of destabilizing local tissue homeostasis in a patient-dependent manner [20,21]. Within this context, oxidative stress has emerged as a plausible and unifying mechanism linking material degradation to adverse clinical outcomes.

### 2.2. Particle and Ion Release from Ti-6Al-4V Implants

Dental implants are subject to continuous chemical and mechanical challenges throughout their clinical lifespan. Corrosion, tribocorrosion, and mechanical wear at the implant surface and implant–abutment interface inevitably result in the release of metal particles and ions into surrounding tissues. These processes are further exacerbated by inflammatory conditions, acidic microenvironments, and mechanical micromotion, all of which are common in the peri-implant niche [22]. Multiple experimental and clinical studies have demonstrated the presence of titanium-containing particles in peri-implant soft tissues, alveolar bone, and regional lymph nodes, confirming their capacity to migrate beyond the immediate implant surface [14,16,21]. In the case of Ti-6Al-4V alloys, released degradation products may additionally contain aluminum and vanadium species, which differ from titanium in their redox activity and biological reactivity. Notably, nanoscale and microscale particles exhibit a high surface-to-volume ratio, enhancing their potential to participate in redox reactions and to interact with surrounding cells [23,24]. Importantly, smaller titanium-containing particles are more readily internalized by peri-implant cells and have been detected beyond the local implant site, including in regional lymph nodes and systemic circulation, indicating their capacity for tissue penetration and systemic dissemination [14,21].

Rather than being biologically inert, these particles and ions represent active sources of oxidative challenge. Their interaction with immune and stromal cells promotes reactive oxygen species generation, either directly through surface-mediated redox reactions or indirectly via activation of cellular ROS-producing systems. Consequently, material degradation constitutes an upstream event capable of initiating redox imbalance within peri-implant tissues. Taken together, these material degradation processes constitute multiple, converging sources of oxidative stress at the implant–tissue interface, as summarized in Table 1.

### 2.3. Oxidative Stress as a Primary Amplifier of Peri-Implant Inflammation

Peri-implant inflammation has traditionally been conceptualized as a biofilm-driven process analogous to periodontitis. While microbial factors undoubtedly play a critical role, this model alone fails to explain the variability and persistence of inflammatory responses observed in certain implant cases. Emerging evidence indicates that oxidative stress acts as a key amplifier of peri-implant inflammation by lowering the threshold for immune activation and sustaining inflammatory signaling [25,26]. Excessive ROS generation in response to implant-derived particles enhances the secretion of pro-inflammatory mediators such as tumor necrosis factor-α and interleukin-1β, while simultaneously impairing regulatory and reparative pathways. Importantly, oxidative stress does not merely accompany inflammation but actively shapes its magnitude and duration. ROS-sensitive signaling pathways modulate macrophage behaviour, cytokine production, and inflammasome activation, thereby reinforcing a pro-inflammatory microenvironment even in the absence of high bacterial burden [27]. This redox-driven amplification helps to explain why titanium particles frequently act synergistically with bacterial components rather than as isolated inflammatory triggers. Mechanistically, elevated ROS levels lower the activation threshold of pattern recognition receptors, such as TLR4, thereby sensitizing innate immune cells to otherwise subthreshold microbial and material-derived signals. In such scenarios, oxidative stress integrates material-derived and microbial stimuli into a unified pathological response, promoting chronic inflammation and tissue breakdown. As a result, peri-implant disease progression may be viewed not solely as an infectious process but as a condition in which oxidative imbalance plays a central pathogenic role [28,29].

This framework highlights that peri-implant pathology arises when antioxidant defense systems fail to compensate for continuous material-derived ROS generation, transforming physiological redox signaling into chronic oxidative stress (Table 2).

### 2.4. Implications for Peri-Implant Tissue Homeostasis

The cumulative effects of particle release and oxidative stress extend beyond inflammation to fundamental processes governing tissue maintenance and regeneration. Elevated ROS levels disrupt osteogenic differentiation of mesenchymal progenitor cells, enhance osteoclastogenesis, and degrade extracellular matrix components critical for soft tissue integrity. Collectively, these effects shift peri-implant tissue dynamics from regeneration toward degeneration [39]. Importantly, oxidative stress feeds back into material degradation by accelerating corrosion and ion release, establishing a self-amplifying pathological loop between implant degradation and tissue inflammation. Crucially, oxidative stress also feeds back into material degradation processes by accelerating corrosion and ion release, thereby establishing a self-perpetuating pathological loop [40,41]. This bidirectional interaction between implant material and host tissues undermines long-term tissue integration and challenges the concept of stable osseointegration as a purely mechanical or structural phenomenon.

Taken together, available evidence supports the view that Ti-6Al-4V dental implants can act as sustained sources of oxidative stress in peri-implant tissues. In susceptible individuals, this redox imbalance constitutes a primary destabilizing factor that precedes and amplifies immune dysregulation, ultimately contributing to adverse clinical outcomes.

## 3. Redox-Sensitive Immune Dysregulation at the Implant–Tissue Interface

### 3.1. Oxidative Stress as a Modulator of Innate Immune Responses

Innate immune mechanisms dominate the early and long-term host response to dental implants and play a critical role in determining whether peri-implant tissues progress toward stable integration or chronic inflammation. Among innate immune cells, macrophages are central regulators of tissue homeostasis, coordinating inflammatory signaling, extracellular matrix remodeling, and bone turnover [42]. Importantly, macrophage behaviour is highly sensitive to the local redox environment. Excessive reactive oxygen species generation at the implant–tissue interface fundamentally alters innate immune signaling. Oxidative stress modulates transcriptional and post-translational pathways involved in cytokine production, phagocytic activity, and inflammasome assembly [30,43,44]. Rather than acting as a secondary consequence of inflammation, redox imbalance directly shapes the magnitude and persistence of innate immune activation in peri-implant tissues. In the context of Ti-6Al-4V implants, material-derived particles and ions contribute to a sustained oxidative milieu that favors pro-inflammatory immune responses. This redox-driven modulation of innate immunity provides a mechanistic link between material degradation and chronic inflammatory outcomes observed in a subset of implant patients [45].

### 3.2. Macrophage Polarization Under Oxidative Conditions

Macrophage polarization represents a dynamic spectrum rather than a binary state; however, a functional distinction between pro-inflammatory (M1-like) and pro-regenerative (M2-like) phenotypes remains useful for understanding implant–tissue interactions [46]. Physiological wound healing following implant placement requires a timely transition from an initial inflammatory phase toward a regenerative, resolution-oriented immune profile. Oxidative stress disrupts this transition. Elevated ROS levels promote sustained M1-like macrophage polarization, characterized by increased production of pro-inflammatory cytokines, nitric oxide, and additional reactive oxygen species. This phenotype reinforces inflammatory signaling while simultaneously inhibiting pathways associated with tissue repair and osteogenesis [47,48]. Importantly, redox imbalance stabilizes pro-inflammatory macrophage states, making resolution of inflammation less likely even after the initial surgical insult has subsided. In this context, failure of the physiological M1-to-M2 macrophage transition emerges as a hallmark of redox-driven implant intolerance rather than a transient inflammatory response. In peri-implant tissues exposed to Ti-6Al-4V degradation products, macrophages are repeatedly challenged by both material-derived and microbial stimuli. Oxidative stress integrates these signals into a persistent inflammatory phenotype, thereby linking environmental stressors to immune dysregulation without requiring antigen-specific adaptive immune responses [31].

### 3.3. Inflammasome Activation as a Redox-Dependent Process

Among redox-sensitive immune pathways, inflammasome signaling has attracted increasing attention in the context of implant-related inflammation. The NLRP3 inflammasome, in particular, responds to a wide range of danger-associated signals, including particulate matter, ionic fluxes, and oxidative stress. ROS play a permissive and amplifying role in inflammasome assembly and activation, facilitating caspase-1 activation and the maturation of interleukin-1β [31,32,33].

Titanium alloy-derived particles have been shown to activate inflammasome pathways in macrophages and other immune cells, an effect that is strongly potentiated under oxidative conditions. This redox dependency provides a mechanistic explanation for the chronic and self-sustaining nature of peri-implant inflammation observed in some patients. Once established, inflammasome-driven cytokine release further enhances oxidative stress and immune activation, reinforcing a pathological feedback loop [34,35,36].

Crucially, inflammasome activation in this context does not require adaptive immune sensitization. Instead, it reflects a redox-mediated danger response to persistent material-derived and environmental stressors at the implant–tissue interface [49].

### 3.4. Immune Intolerance Versus Metal Allergy: A Redox-Based Perspective

The predominance of redox-sensitive innate immune mechanisms challenges the widespread interpretation of adverse reactions to titanium implants as manifestations of classical metal allergy [50]. Delayed-type hypersensitivity reactions are mediated by antigen-specific T lymphocytes and require prior sensitization, a mechanism that is inconsistently supported in patients with implant-related complications [51,52].

In contrast, the concept of immune intolerance more accurately captures the biological processes described herein. Immune intolerance encompasses sustained innate immune activation, oxidative stress, and impaired tissue adaptation in response to continuous material-derived challenges. Within this framework, oxidative stress acts as the primary destabilizing factor, while immune dysregulation represents a downstream effector process [53,54,55].

Recognizing this distinction has important implications for both diagnosis and clinical decision-making. Conventional allergy testing fails to detect redox-driven pathology and may therefore underestimate the contribution of implant materials to adverse tissue responses. A redox-centered interpretation provides a more coherent explanation for negative allergy tests in patients who nevertheless exhibit persistent peri-implant inflammation or implant failure.

To clarify the mechanistic distinction between classical metal allergy and redox-driven implant intolerance, the key biological and clinical features of both entities are summarized in Table 3.

These distinct therapeutic implications further underscore why redox-driven implant intolerance cannot be managed using diagnostic and treatment paradigms developed for classical metal allergy.

### 3.5. Integration with Redox-Centered Pathogenesis

When viewed in conjunction with the oxidative mechanisms outlined in Section 2, immune dysregulation emerges as an integral component of a broader redox-centered pathogenic cascade. Material degradation initiates oxidative stress, which in turn modulates innate immune responses, sustains inflammation, and disrupts tissue regeneration. This integrated perspective reinforces the concept that adverse tissue responses to Ti-6Al-4V implants are not isolated immunological anomalies but predictable outcomes of sustained redox imbalance in susceptible hosts.

## 4. Alloy Composition and Redox Burden of Ti-6Al-4V Implants

### 4.1. Rationale for the Use of Ti-6Al-4V in Dental Implants

The Ti-6Al-4V alloy has been widely adopted in dental implantology due to its superior mechanical properties compared with commercially pure titanium, including increased tensile strength, fatigue resistance, and fracture toughness [56]. These characteristics are particularly advantageous in load-bearing clinical situations and in patients exposed to high occlusal forces. However, the enhanced mechanical performance of Ti-6Al-4V is achieved through the incorporation of aluminum and vanadium, elements that fundamentally alter not only the physical but also the biological behaviour of the material [57].

From a biological perspective, alloying introduces heterogeneity in surface chemistry and electrochemical stability. Under physiological conditions, titanium forms a protective oxide layer that contributes to corrosion resistance. In contrast, the presence of aluminum- and vanadium-containing phases may create localized electrochemical differences at the implant surface, particularly under inflammatory or acidic conditions [58,59,60]. These microenvironmental changes have important implications for redox balance at the implant–tissue interface.

### 4.2. Aluminum and Vanadium as Modulators of Oxidative Stress

Aluminum and vanadium are not biologically inert elements. Experimental studies in various biological systems have demonstrated that both metals can interfere with cellular redox homeostasis, mitochondrial function, and oxidative signaling pathways. Aluminum exposure has been associated with increased oxidative stress, lipid peroxidation, and impairment of antioxidant defense mechanisms, while vanadium compounds are known to participate in redox cycling and to modulate phosphate- and redox-sensitive enzymatic pathways [37,38,61].

In the context of Ti-6Al-4V dental implants, corrosion and wear processes may lead to the release of aluminum- and vanadium-containing ions and particles into peri-implant tissues. Once internalized by resident or infiltrating cells, these species can contribute to mitochondrial dysfunction and excessive reactive oxygen species production. Importantly, their redox activity may amplify oxidative stress beyond that induced by titanium alone, thereby increasing the biological burden at the implant–tissue interface [38,62].

### 4.3. Alloy-Derived Particles and Redox-Sensitive Cellular Responses

The biological behaviour of alloy-derived particles differs qualitatively from that of bulk titanium. Nanoscale and microscale debris generated from Ti-6Al-4V exhibits increased surface reactivity and solubility, enhancing its capacity to engage in redox reactions and to activate cellular stress pathways [63]. In vitro studies have shown that macrophages exposed to Ti-6Al-4V particles generate higher levels of reactive oxygen species and pro-inflammatory mediators compared with cells exposed to particles derived from commercially pure titanium [38,62,63].

These observations suggest that adverse tissue responses attributed to “titanium intolerance” may, in some cases, reflect a broader intolerance to alloy-derived oxidative stress rather than to titanium itself [64]. From a redox biology perspective, the alloy composition becomes a critical determinant of the magnitude and persistence of oxidative imbalance, particularly in tissues already challenged by inflammation or mechanical stress [65].

### 4.4. Implications for Patient Susceptibility and Material Selection

Host-related factors further modulate the biological impact of alloy-derived oxidative stress. Patients with pre-existing immune dysregulation, chronic inflammatory conditions, or impaired antioxidant capacity may exhibit a reduced ability to buffer redox challenges imposed by implant materials. In such individuals, exposure to aluminum- and vanadium-containing degradation products may more readily tip the balance toward pathological oxidative stress and immune dysregulation [9,17,66].

These considerations challenge the assumption that all titanium-based implants are biologically equivalent and underscore the importance of alloy composition in material selection. While Ti-6Al-4V offers undeniable mechanical advantages, its redox implications warrant careful consideration, particularly in patients with increased susceptibility to oxidative stress–driven tissue responses [67]. Integrating redox awareness into biomaterial choice represents a rational step toward more individualized and biologically informed implant therapy.

**Figure 2:**
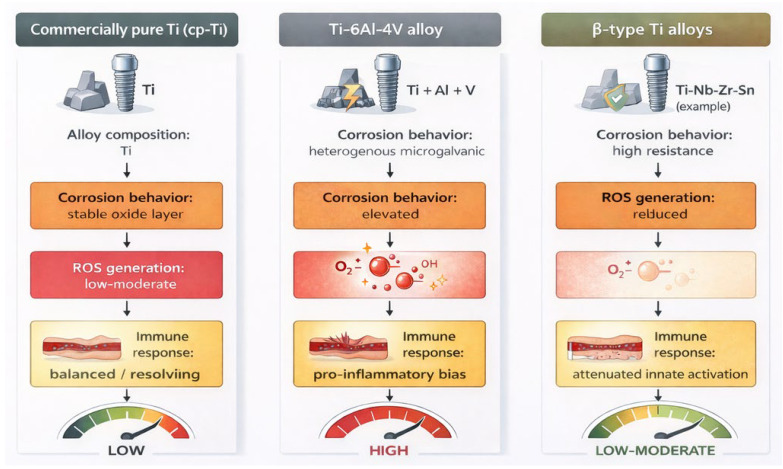
Comparative redox-related biological profiles of titanium-based implant materials. Differences in alloy composition influence corrosion behavior, reactive oxygen species (ROS) generation, and downstream immune responses at the implant–tissue interface. Compared with commercially pure titanium (cp-Ti), Ti-6Al-4V alloys introduce aluminum- and vanadium-containing degradation products that may amplify oxidative and inflammatory burden, whereas emerging β-type titanium alloys demonstrate improved corrosion resistance and reduced redox-driven innate immune activation in preclinical studies. Created in BioRender. Mierzejewska, A. (12 February 2026) https://BioRender.com/hwwyury.

From a redox biology perspective, titanium-based implant materials are not biologically equivalent. Commercially pure titanium (cp-Ti) exhibits relatively stable corrosion behaviour and a lower intrinsic redox burden, whereas Ti-6Al-4V alloys introduce aluminum and vanadium containing degradation products that can amplify oxidative stress and innate immune activation. In contrast, emerging β-type titanium alloys, typically free of Al and V, demonstrate improved corrosion resistance and reduced ROS-mediated cellular stress in preclinical models, suggesting a potentially more favorable redox profile for susceptible patients (Figure 2).

## 5. Diagnostic Challenges in Identifying Redox-Driven Implant Intolerance

### 5.1. Limitations of Conventional Allergy-Based Diagnostic Approaches

The diagnosis of adverse tissue responses to titanium-based dental implants remains a significant clinical challenge. In routine practice, suspected material-related complications are often evaluated using diagnostic tools developed for classical metal allergy, such as patch testing or lymphocyte transformation tests [68,69]. However, these approaches primarily assess antigen-specific adaptive immune responses and therefore fail to capture the biological mechanisms increasingly implicated in implant-related complications.

Clinical experience and experimental evidence consistently demonstrate a poor correlation between positive allergy test results and implant outcomes. Conversely, many patients presenting with persistent peri-implant inflammation, impaired healing, or unexplained implant failure exhibit negative results in standard allergy testing. This diagnostic discrepancy reflects a fundamental mismatch between the mechanisms being tested and the underlying pathophysiology of many implant-related adverse reactions [70,71,72].

### 5.2. Redox-Driven Pathology Beyond the Scope of Allergy Testing

As outlined in preceding sections, oxidative stress represents a central driver of tissue dysregulation at the implant–tissue interface. Redox imbalance promotes immune activation, impairs regenerative processes, and sustains inflammation independently of antigen-specific sensitization. These processes are inherently invisible to allergy-based diagnostic frameworks, which are not designed to detect disturbances in redox homeostasis or innate immune signaling [9,45,73].

Importantly, oxidative stress–driven pathology may develop gradually and remain subclinical for extended periods, further complicating its recognition. By the time overt clinical manifestations such as peri-implant bone loss or implant instability become apparent, redox imbalance and immune dysregulation may already be well established. The absence of diagnostic tools capable of detecting early redox disturbances therefore contributes to delayed or incomplete identification of material-related risk factors [74,75].

### 5.3. Toward Redox-Aware Diagnostic Strategies

The recognition of oxidative stress as a key determinant of implant–tissue interactions highlights the need for diagnostic strategies that extend beyond conventional allergy testing. Candidate biomarkers reflecting oxidative and nitrosative stress include DNA oxidation markers such as 8-hydroxy-2′-deoxyguanosine (8-OHdG), lipid peroxidation products such as malondialdehyde (MDA), and protein modifications including nitrotyrosine. Assessment of such markers in peri-implant crevicular fluid, saliva, or systemic circulation may provide indirect insight into redox imbalance at the implant–tissue interface, although clinical validation is still limited. Potential approaches include the assessment of local or systemic markers of oxidative stress, evaluation of inflammatory and redox-sensitive cytokine profiles, and functional assays examining macrophage responses to implant-derived particles under controlled redox conditions. At present, such methods remain largely confined to experimental or translational research settings and are not standardized for routine clinical use. Nevertheless, they offer a conceptual framework for future diagnostic development [76,77,78]. Integrating redox biology into diagnostic thinking may enable more accurate identification of patients at increased risk for implant-related complications and facilitate earlier, more targeted clinical decision-making.

### 5.4. Clinical Implications of Diagnostic Limitations

The inability to detect redox-driven implant intolerance using existing diagnostic tools has important clinical consequences. Negative allergy test results are often interpreted as evidence that implant materials cannot be implicated in adverse outcomes, reinforcing a narrow focus on mechanical or microbiological explanations. This interpretation may delay appropriate intervention and contribute to repeated treatment failure in susceptible individuals. A redox-centered diagnostic perspective does not imply routine screening or indiscriminate testing but rather emphasizes informed clinical judgment [15,21,79]. Awareness of oxidative stress–driven mechanisms may assist clinicians in recognizing patterns of implant failure that fall outside conventional etiological categories. Ultimately, improving diagnostic sensitivity to redox imbalance represents a critical step toward more biologically informed and patient-specific implant therapy [80].

## 6. Clinical Implications and Redox-Aware Perspectives in Implant Dentistry

### 6.1. Rethinking Implant-Related Complications Through a Redox Lens

Implant-related complications are traditionally interpreted within mechanical and microbiological frameworks, with primary emphasis placed on surgical technique, occlusal loading, and plaque control. While these factors remain fundamental determinants of implant success, exclusive reliance on them does not adequately explain a subset of clinical scenarios characterized by impaired healing, persistent peri-implant inflammation, or early implant failure occurring under otherwise optimal clinical conditions. Increasing clinical and experimental evidence indicates that, in such cases, adverse outcomes may arise despite appropriate implant positioning, adequate load distribution, and effective plaque control [6,25].

From a clinical standpoint, recognizing oxidative stress as a biologically relevant modifier of peri-implant tissue tolerance offers a more nuanced framework for interpreting these otherwise unexplained complications. Rather than attributing failure exclusively to technical shortcomings or microbial burden, this perspective highlights the possibility that peri-implant tissues in certain patients exhibit a reduced capacity to adapt to continuous material-derived challenges. In this context, implant failure may reflect a mismatch between the oxidative burden imposed by the implant material and the host’s ability to maintain redox homeostasis, potentially reflecting impaired antioxidant responsiveness, leading to sustained inflammation and impaired tissue integration [26,81].

This interpretation aligns with contemporary views that peri-implant diseases are multifactorial conditions shaped by the interaction between microbial, mechanical, material-related, and host-specific factors. Recent consensus reports and clinical guidelines increasingly acknowledge that host inflammatory status, systemic comorbidities, and biological responses to implant materials can modulate disease susceptibility and progression independently of plaque accumulation alone. Importantly, a redox-centered clinical lens does not negate established risk factors but complements them, providing a biologically coherent explanation for patient-specific heterogeneity in implant outcomes.

Clinically, this approach encourages practitioners to consider redox-related susceptibility when peri-implant inflammation or bone loss persists despite adherence to established preventive and therapeutic protocols. In such cases, continued escalation of mechanical or antimicrobial interventions alone may be insufficient, as the underlying driver may lie in sustained biological stress rather than ongoing external insult. Incorporating redox awareness into clinical reasoning therefore represents a shift toward a more integrative and patient-specific interpretation of implant-related complications, without implying immediate changes to standard treatment algorithms.

### 6.2. Implications for Patient Assessment and Risk Stratification

Although routine pre-implant screening for oxidative stress or material intolerance is not currently supported by sufficient clinical evidence, heightened awareness of redox-related risk factors may improve clinical judgment. From a redox biology perspective, several patient-related conditions may predispose peri-implant tissues to impaired oxidative stress buffering and heightened immune dysregulation. Smoking is associated with increased systemic and local ROS generation, reduced antioxidant enzyme activity, and compromised microvascular function, collectively amplifying oxidative burden at the implant site. Similarly, diabetes mellitus is characterized by chronic oxidative stress, mitochondrial dysfunction, and impaired wound healing capacity, which may exacerbate redox-driven inflammatory responses to implant-derived degradation products.

Chronic inflammatory and autoimmune disorders further contribute to baseline immune activation and persistent oxidative imbalance, potentially lowering the threshold for material-induced tissue intolerance. In addition, individuals with reduced antioxidant responsiveness—whether due to metabolic conditions, aging, or genetic variability in redox-regulating pathways such as Nrf2—may exhibit limited capacity to restore redox homeostasis following implant placement.

Clinically, these risk profiles may help identify patients in whom standard titanium alloy implants impose a disproportionate biological burden despite optimal surgical technique and plaque control. In such individuals, heightened vigilance, individualized material selection, and closer long-term monitoring may be warranted.

Patients with chronic inflammatory diseases, autoimmune conditions, metabolic disorders, or known impairments in antioxidant defense mechanisms may exhibit increased vulnerability to oxidative stress–driven tissue responses. Consistent with this view, peri-implant disease is increasingly regarded as a multifactorial condition in which host-related inflammatory status, systemic comorbidities, and implant material characteristics may modulate tissue tolerance and disease progression, even under adequate plaque control. While routine redox screening is not yet clinically available, awareness of these risk profiles may assist clinicians in recognizing patient-specific vulnerability to redox-driven peri-implant complications.

In such cases, implant-related complications should not be automatically attributed to technical failure or inadequate oral hygiene. Instead, clinicians may consider the possibility that sustained oxidative stress and redox-sensitive immune dysregulation contribute to compromised tissue integration. This clinical pattern aligns with reports demonstrating elevated oxidative and nitrosative stress biomarkers in peri-implant fluids and systemic circulation, even in the absence of positive allergy testing [18,20,21]. Importantly, negative results in conventional allergy testing should not be interpreted as definitive exclusion of biologically relevant material–tissue interactions. In clinical practice, redox-driven implant intolerance may be suspected when peri-implant inflammation or bone loss persists despite optimal plaque control, appropriate implant positioning, and absence of mechanical overload. In such cases, implant material characteristics and host oxidative or inflammatory susceptibility may warrant consideration as contributing factors.

### 6.3. Redox Awareness in Biomaterial Selection and Surface Design

Recognition of oxidative stress as a central determinant of implant–tissue interactions has direct implications for biomaterial selection and implant surface design. Titanium-based implant materials differ substantially in alloy composition, electrochemical stability, and corrosion behaviour, all of which influence the magnitude of oxidative challenge imposed on peri-implant tissues. From a redox perspective, these material-dependent differences are not merely physicochemical variables but biologically meaningful determinants of long-term tissue tolerance [41,45].

Ti-6Al-4V alloys, while offering superior mechanical performance, introduce aluminum- and vanadium-containing phases that may alter local electrochemical conditions, particularly under inflammatory or acidic microenvironments. Experimental studies demonstrate that alloy heterogeneity, microgalvanic coupling, and tribocorrosion phenomena can accelerate particle and ion release, thereby amplifying reactive oxygen species generation and redox-sensitive cellular stress responses. In contrast, commercially pure titanium and emerging β-type titanium alloys lacking aluminum and vanadium have shown improved corrosion resistance and a reduced propensity to induce oxidative and inflammatory signaling in preclinical models [60]. These findings suggest that alloy composition constitutes a modifiable determinant of redox burden, particularly relevant in patients with heightened susceptibility to oxidative stress.

Beyond bulk composition, surface characteristics play a critical role in shaping redox dynamics at the implant–tissue interface. Surface roughness, oxide layer stability, and susceptibility to corrosion influence both the quantity and the biological reactivity of released degradation products. Surface engineering strategies may substantially limit aluminum and vanadium release from Ti-6Al-4V alloys by enhancing oxide layer stability and corrosion resistance. Techniques such as controlled anodization, formation of thickened TiO_2_ barriers, ceramic or bioactive coatings, and tribocorrosion-resistant surface modifications have been shown to reduce ion dissolution and particle generation under inflammatory and acidic conditions. By minimizing alloy degradation, these treatments may indirectly attenuate ROS generation and redox-driven immune activation. From a redox perspective, improved surface stability represents a modifiable determinant of biological burden imposed by Ti-6Al-4V implants, particularly in susceptible patients. Surface engineering strategies aimed at enhancing corrosion resistance and limiting particle release—such as controlled anodization, stable oxide coatings, and surface treatments that minimize tribocorrosion—have demonstrated favorable effects on cellular responses in vitro and in animal models. Importantly, several surface modifications have been shown to influence not only ROS generation but also redox-sensitive signaling pathways, including Nrf2-mediated antioxidant responses in peri-implant cells.

More recently, redox-modulating surface concepts have emerged, including coatings designed to scavenge reactive oxygen species or to respond dynamically to oxidative microenvironments. Although these approaches remain largely experimental, early evidence suggests that redox-responsive surfaces can influence immune cell behaviour and support osteogenic processes under conditions of oxidative challenge. While long-term clinical data are currently lacking, these strategies exemplify a shift toward biologically informed implant design that accounts for the dynamic oxidative environment of peri-implant tissues [9,10].

From a clinical perspective, redox awareness in biomaterial selection does not imply routine avoidance of Ti-6Al-4V implants or indiscriminate adoption of novel materials. Rather, it supports a more individualized approach in which implant material properties are considered alongside patient-related factors such as inflammatory burden, systemic comorbidities, and potential impairment of antioxidant defenses. In susceptible individuals, minimizing material-derived oxidative stress through informed material choice and surface design may contribute to improved tissue adaptation and long-term implant stability.

### 6.4. Toward a Redox-Informed Clinical Paradigm

The translational implications of redox-driven implant intolerance, spanning material selection, surface engineering, diagnostics, and patient-related factors, are summarized in Table 4.

Integrating redox biology into implant dentistry represents a conceptual shift rather than an immediate change in clinical protocols. By acknowledging oxidative stress as a central, mechanistically coherent factor in adverse tissue responses, clinicians and researchers can move beyond oversimplified etiological models and toward a more nuanced understanding of implant biocompatibility.

Surface engineering strategies aimed at reducing corrosion, particle release, and oxidative stress—such as controlled anodization, anti-corrosive coatings, and nanostructured surfaces—have demonstrated favorable effects on cellular responses and early osseointegration in preclinical and translational studies. Although long-term clinical evidence remains limited, emerging data suggest that surface modifications can modulate inflammatory signaling and improve tissue responses, supporting their consideration within a redox-aware implant design framework [71,81].

This redox-informed paradigm emphasizes the dynamic interaction between material properties and host biology, highlighting the importance of patient-specific susceptibility and long-term tissue adaptation. Ultimately, incorporating redox considerations into clinical reasoning may contribute to improved implant outcomes and more personalized treatment strategies.

Within this paradigm, oxidative stress is not viewed as a secondary epiphenomenon but as a central determinant of long-term implant–tissue compatibility, linking material properties with host-specific biological resilience. At present, most redox-modulating strategies remain supported primarily by preclinical and early translational evidence, underscoring the need for well-designed longitudinal clinical studies.

## 7. Discussion

This critical review reframes adverse tissue responses to Ti-6Al-4V dental implants as a redox-driven pathological process rather than as isolated immunological or allergic phenomena. While titanium-based implants remain highly successful in the majority of clinical scenarios, the evidence synthesized herein demonstrates that, in susceptible individuals, implant materials can destabilize peri-implant tissue homeostasis through sustained oxidative stress. Importantly, this oxidative burden arises not merely from transient surgical trauma or secondary inflammation but from continuous material degradation processes that persist throughout the implant’s functional lifetime.

### 7.1. Oxidative Stress Versus Antioxidant Capacity: Why Redox Balance Matters

A key conceptual point emerging from this analysis is that oxidative stress should not be equated simply with the presence of reactive oxygen species. In physiological contexts, controlled ROS generation is indispensable for wound healing, angiogenesis, and osseointegration. Pathology arises when ROS production exceeds the buffering capacity of local antioxidant defense systems. Thus, adverse peri-implant tissue responses are best understood as a failure of redox balance rather than as a consequence of ROS exposure per se.

Peri-implant tissues possess intrinsic antioxidant systems—including enzymatic pathways such as superoxide dismutases, catalase, glutathione peroxidases, and redox-sensitive transcriptional regulators such as the Nrf2–Keap1 axis—that normally constrain oxidative signaling and promote resolution of inflammation. Persistent exposure to implant-derived particles and ions may overwhelm or dysregulate these systems, particularly in tissues already challenged by inflammation, mechanical stress, or compromised vascularization. From this perspective, oxidative stress reflects not only increased ROS generation but also insufficient antioxidant responsiveness, a distinction that is central to the scope of *Antioxidants*.

Crucially, current implant-related diagnostic and therapeutic paradigms rarely consider antioxidant capacity as a determinant of tissue tolerance. This omission may partly explain the heterogeneity of clinical outcomes observed with ostensibly identical implant systems. Patients who are unable to mount an adequate antioxidant response may be predisposed to redox-driven immune dysregulation and impaired tissue adaptation, even in the absence of classical risk factors.

### 7.2. Redox-Driven Immune Dysregulation Beyond the Allergy Paradigm

The redox-centered framework presented here also clarifies why conventional allergy-based interpretations fail to account for many implant-related complications. Classical delayed-type hypersensitivity reactions depend on antigen-specific adaptive immune mechanisms, whereas the dominant processes described in peri-implant tissues involve innate immune activation modulated by oxidative stress. Macrophage polarization, inflammasome signaling, and cytokine release are all highly sensitive to redox cues and can be sustained independently of immune sensitization.

By emphasizing oxidative stress as an upstream regulator, this model reconciles negative allergy test results with persistent clinical inflammation. Rather than reflecting immunological tolerance, such findings may indicate that pathology resides outside the scope of adaptive immune diagnostics. The concept of immune intolerance driven by redox imbalance more accurately captures the continuous, non-specific nature of the host response to implant-derived degradation products.

### 7.3. Alloy Composition as a Determinant of Redox Burden

An important implication of this review is that not all titanium-based implants impose equivalent redox challenges. The Ti-6Al-4V alloy, while mechanically advantageous, introduces aluminum and vanadium species with distinct redox activities that may amplify oxidative stress beyond that associated with commercially pure titanium. Alloy heterogeneity, microgalvanic effects, and differential corrosion behavior under inflammatory conditions can collectively increase ROS generation and cellular stress responses.

These considerations suggest that adverse reactions attributed generically to “titanium intolerance” may, in some cases, reflect a broader intolerance to sustained alloy-derived oxidative burden. Recognizing alloy composition as a biologically relevant variable challenges the assumption of universal equivalence among titanium implants and supports a more nuanced, redox-informed approach to biomaterial selection.

### 7.4. Translational Implications: Toward Redox-Aware Implant Design and Assessment

Although this review does not advocate immediate changes to clinical protocols, it highlights several translational directions with relevance to implant dentistry and biomaterials research. First, surface engineering strategies aimed at minimizing corrosion, particle release, and tribocorrosion may reduce the oxidative burden imposed on peri-implant tissues. Second, emerging approaches that incorporate redox-modulating or antioxidant-responsive surface features warrant further investigation, particularly in patients with heightened susceptibility to oxidative stress.

From a diagnostic standpoint, the recognition of redox imbalance as a central pathogenic factor underscores the limitations of current allergy-based testing. While routine assessment of oxidative stress markers is not yet feasible in clinical practice, future diagnostic frameworks may benefit from integrating redox-sensitive biomarkers, functional immune assays, or localized measures of oxidative and antioxidant status. Such tools could improve patient risk stratification and help identify individuals for whom conventional implant materials pose a higher biological burden.

### 7.5. Limitations and Future Directions

Several limitations must be acknowledged. Much of the mechanistic evidence supporting redox-driven implant intolerance derives from in vitro and animal models, and direct clinical quantification of oxidative stress at the implant–tissue interface remains limited. Furthermore, the dynamic interplay between ROS generation and antioxidant defense systems in peri-implant tissues has not been systematically characterized in longitudinal human studies.

Future research should aim to define redox thresholds that distinguish physiological adaptation from pathological intolerance, to identify host-related factors that impair antioxidant responsiveness, and to evaluate whether redox-modulating material strategies translate into improved long-term clinical outcomes. Addressing these gaps will be essential for moving from conceptual frameworks toward actionable clinical insights.

### 7.6. Integrating Redox Biology into the Concept of Biocompatibility

Taken together, the findings discussed in this review support a shift from static definitions of biocompatibility toward a dynamic, redox-informed understanding of host–material interactions. Rather than assuming biological neutrality, implant materials should be evaluated in terms of the oxidative and antioxidant demands they impose on surrounding tissues over time. Within this paradigm, oxidative stress emerges not as an incidental byproduct but as a central determinant of tissue response, immune regulation, and long-term implant stability.

## 8. Conclusions

Ti-6Al-4V dental implants cannot be considered biologically inert in all clinical contexts. Evidence reviewed in this article indicates that continuous material degradation may impose a sustained oxidative burden on peri-implant tissues, leading to disruption of redox homeostasis and compromised tissue integration in susceptible individuals. Importantly, oxidative stress in this setting reflects not only increased reactive oxygen species generation but also insufficient local antioxidant capacity to counter persistent redox challenges.

This redox-centered framework provides a coherent explanation for peri-implant inflammation, impaired healing, and implant failure that occur in the absence of classical mechanical or microbiological risk factors. Rather than representing rare allergic reactions, many adverse responses to titanium implants appear to arise from redox-sensitive innate immune dysregulation driven by sustained oxidative imbalance. The alloy composition of Ti-6Al-4V, including aluminum- and vanadium-containing degradation products, may further amplify this oxidative burden.

Recognizing oxidative stress as a central determinant of implant–tissue interactions highlights the need to move beyond allergy-based interpretations toward redox-aware concepts of biocompatibility. Integrating redox biology into biomaterial selection, surface engineering, and future diagnostic strategies may ultimately improve patient-specific implant outcomes. This redox-centered perspective also supports future exploration of redox- and antioxidant-informed strategies to improve long-term implant–tissue compatibility. Importantly, recognition of oxidative stress as a central determinant of implant–tissue interactions also opens the perspective that modulation of the local redox environment may represent a complementary strategy to improve osseointegration. Antioxidant agents—whether delivered systemically, locally, or incorporated into implant surface modifications—may enhance peri-implant tissue resilience by restoring redox balance and supporting physiological bone remodeling. By attenuating excessive reactive oxygen species (ROS) signaling, antioxidant approaches may facilitate the transition from pro-inflammatory macrophage activation toward a pro-regenerative immune phenotype, thereby promoting stable bone–implant integration.

Experimental studies have demonstrated that antioxidant molecules, redox-responsive coatings, and ROS-scavenging biomaterials can improve osteogenic differentiation, reduce inflammatory cytokine release, and enhance early bone formation under conditions of oxidative challenge. Although robust clinical evidence remains limited, these findings suggest that antioxidant-based strategies may represent a biologically rational adjunct to conventional implant therapy, particularly in patients with increased susceptibility to oxidative stress–driven tissue dysregulation. Future research should aim to clarify optimal delivery methods, dosing, and long-term clinical impact of antioxidant interventions in implant dentistry.

## Figures and Tables

**Table 1:** Sources and biological consequences of oxidative stress associated with Ti-6Al-4V dental implants [24,25,21,22].

Source of Oxidative Stress	Origin at Implant–Tissue Interface	Primary Redox-Related Effects	Antioxidant Systems Affected	Downstream Biological Consequences
Metal particle release	Corrosion, tribocorrosion, wear	Surface-mediated ROS generation	↓ GSH, ↓ SOD	Cellular oxidative damage
Metal ion release (Ti, Al, V)	Alloy degradation	Mitochondrial ROS production	Nrf2 dysregulation	Impaired osteogenic differentiation
Acidic microenvironment	Inflammation, biofilm	Enhanced redox reactions	Catalase exhaustion	Increased corrosion and ion release
Mechanical micromotion	Implant–abutment interface	Activation of ROS-producing enzymes	Redox imbalance	Sustained local oxidative stress

↓ indicates a decrease in the level or activity of the parameter compared with the control group

**Table 2:** Failure of antioxidant defense systems in redox-driven peri-implant tissue pathology [30,31,32,33,34,35,36,37,38].

ROS Source/Redox Trigger	Primary Antioxidant System Involved	Point of Antioxidant Failure	Resulting Redox Imbalance	Downstream Biological Outcome
Titanium and alloy particle release	Glutathione system (GSH/GSSG)	GSH depletion due to chronic ROS scavenging	Loss of intracellular redox buffering	Impaired osteogenic differentiation; cellular oxidative damage
Metal ion release (Ti, Al, V)	Mitochondrial antioxidant enzymes (SOD2, GPx)	Mitochondrial antioxidant exhaustion	Mitochondrial ROS amplification	Macrophage pro-inflammatory polarization; inflammasome priming
Persistent inflammatory microenvironment	Catalase and peroxiredoxins	Enzymatic saturation under sustained H_2_O_2_ load	Accumulation of hydrogen peroxide	Extracellular matrix degradation; impaired soft tissue healing
Chronic oxidative signaling	Nrf2–Keap1 pathway	Insufficient or dysregulated Nrf2 activation	Failure of adaptive antioxidant gene expression	Sustained innate immune activation; chronic inflammation
Tribocorrosion and mechanical micromotion	Integrated cellular antioxidant network	Inability to restore redox homeostasis	Persistent redox imbalance	Progressive tissue intolerance and osseointegration failure

**Table 3:** Oxidative stress–driven implant intolerance versus classical metal allergy.

Feature	Redox-Driven Intolerance	Metal Allergy
Immune arm	Innate immunity	Adaptive immunity
Key driver	ROS > antioxidant capacity	Antigen sensitization
Cells involved	Macrophages, neutrophils	T lymphocytes
Diagnostics	No standard tests	Patch/LTT
Allergy tests	Often negative	Often positive
Clinical course	Chronic, progressive	Episodic
Therapeutic implication	Material avoidance, modification, or replacement; focus on reducing oxidative burden	Allergen avoidance or desensitization strategies

**Table 4:** Translational implications of redox-driven implant intolerance in implant dentistry.

Level	Strategy	Redox Rationale	Evidence Level	Current Feasibility
Material	Alternative Ti alloys (e.g., β-Ti)	Adaptive Lower intrinsic redox activity	Preclinical/ early clinical	Limited
	Avoidance of Al/V-containing alloys in susceptible patients	Reduced oxidative burden	Experimental	Limited
Surface	Anti-corrosive coatings	Reduced particle and ion release	Preclinical	Moderate
	Redox-modulating or antioxidant-responsive surfaces	Attenuation of ROS signaling	Experimental	Emerging
Diagnostics	Redox biomarkers (local/systemic)	Early detection of imbalance	Experimental	Low
Patient	Risk stratification based on inflammatory/redox status	Improved patient selection	Conceptual	Moderate

## Data Availability

No new data were created or analyzed in this study. Data sharing is not applicable to this article.
